# Comparison of hematopoietic stem cell transplantation and repeated intensified immunosuppressive therapy as second-line treatment for relapsed/refractory severe aplastic anemia

**DOI:** 10.3389/fimmu.2024.1425076

**Published:** 2024-08-16

**Authors:** Lining Zhang, Jianping Li, Weiru Liang, Xiaoyu Zhang, Shulian Chen, Yuanyuan Shi, Mengze Hao, Xiaoli Zhao, Ming Gong, Jialin Wei, Yi He, Erlie Jiang, Mingzhe Han, Fengkui Zhang, Sizhou Feng

**Affiliations:** ^1^ State Key Laboratory of Experimental Hematology, National Clinical Research Center for Blood Diseases, Haihe Laboratory of Cell Ecosystem, Institute of Hematology and Blood Diseases Hospital, Chinese Academy of Medical Sciences and Peking Union Medical College, Tianjin, China; ^2^ Tianjin Institutes of Health Science, Tianjin, China

**Keywords:** severe aplastic anemia, relapse, refractory, hematopoietic stem cell transplantation, immunosuppressive therapy

## Abstract

The optimal treatment for patients with severe aplastic anemia (SAA) who fail an initial course of antithymocyte globulin (ATG) plus cyclosporine has not yet been established. We compared the effectiveness of allogeneic hematopoietic stem cell transplantation (allo-HSCT) (*n* = 36) with repeated immunosuppressive therapy (IST) (*n* = 33) for relapsed/refractory SAA between 2007 and 2022. In the IST group, patients were retreated with ATG (*n* = 16) or high-dose cyclophosphamide (*n* = 17). The overall response rate was 57.6% at 6 months and 60.6% at 12 months. In the allo-HSCT group, patients received a transplant from a matched sibling donor (*n* = 6), matched unrelated donor (*n* = 7), or haploidentical donor (*n* = 23). All patients achieved neutrophil engraftment, and there were no cases of primary graft failure. The cumulative incidences (CIs) of grades II–IV and III–IV acute graft-versus-host disease (GVHD) were 36.1% ± 0.7% and 13.9% ± 0.3% at day +100, respectively. The 4-year CI of chronic GVHD (cGVHD) was 36.2% ± 0.7%, with moderate to severe cGVHD at 14.9% ± 0.4%. Compared with IST, HSCT recipients showed much higher hematologic recovery rate at 3, 6, and 12 months (63.9%, 83.3%, and 86.1%, respectively, *p* < 0.001). The estimated 4-year overall survival (OS) (79.8% ± 6.8% vs. 80.0% ± 7.3%, *p* = 0.957) was similar; however, the failure-free survival (FFS) was significantly better in the HSCT group (79.8% ± 6.8% vs. 56.6% ± 8.8%, *p* = 0.049). Of note, children in the HSCT cohort were all alive without treatment failures, exhibiting superior OS (100% vs. 50.0% ± 17.7%, *p* = 0.004) and FFS (100% vs. 50.0% ± 17.7%, *p* = 0.004) than children in the IST cohort. Subgroup analysis revealed that younger patients (age ≤ 35 years), especially children, and those with refractory SAA benefited more from HSCT. Therefore, for these patients, salvage HSCT may be more preferable than a second course of IST.

## Introduction

Acquired severe aplastic anemia (SAA) is an immune-mediated hematopoietic stem cell disorder that presents with hypocellular marrow and pancytopenia (1). The first-line treatment options for SAA include allogeneic hematopoietic stem cell transplantation (allo-HSCT) and immunosuppressive therapy (IST) with antithymocyte globulin (ATG) and cyclosporine (CsA). The hematopoietic response rate to IST has been reported to be 70%–80% and the probability of survival at 5 years ranges from 60% to 85% (2–4). Despite improvement with IST, approximately one-third of SAA patients remain refractory to IST (1, 5) and 30%–40% may eventually relapse (3). The management of patients with refractory or relapsed SAA after IST represents a major challenge.

With advances in supportive care, donor selection, and conditioning regimen, the outcomes of alternative donor HSCT in patients with SAA have improved dramatically (6–8). HSCT from matched unrelated donor (MUD) or haploidentical donor (HID) has become an effective salvage treatment for IST failure. Xu et al. reported on the long-term outcomes of 287 SAA patients who underwent salvage HID-HSCT. For previously failed IST, 63 patients received ATG + CsA-based regimens, while the rest received CsA-based regimens. The estimated overall survival (OS) and failure-free survival (FFS) for the whole cohort at 9 years were 85.4% and 84.0%, respectively (9).

Salvage with a repeated course of ATG-based IST has also been employed in some patients. The response rates have varied significantly, ranging from 22% to 77%, and the response in the refractory setting is often inferior to that in the relapsed setting (10–12). Scheinberg et al. summarized the results of rabbit ATG (r-ATG) retreatment in patients with SAA who were refractory to or who had relapsed following horse ATG (h-ATG). The overall response rate (ORR) was 65% in relapsed patients; however, it was only 30% in refractory patients (10).

In addition, high-dose cyclophosphamide (HD-CTX) is highly immunosuppressive and has been used in both treatment-naïve and refractory/relapse SAA (13–16). An advantage of HD-CTX/CsA over ATG/CsA is that it is much cheaper, rendering it a reasonable alternative for those who cannot afford ATG-based therapy. A study reported a large cohort of SAA patients treated with HD-CTX, and confirmed its effectiveness. At 10 years, the OS was 88%, and the response rate was 71% (13). However, a small randomized study (17) combined HD-CTX (200 mg/kg) with CsA for SAA, and found a high rate of fungal infections and early mortality. To reduce the toxicity of CTX, we modified CTX dose to 120 mg/kg and deferred CsA to day 7 after the completion of CTX infusion. Our previous study observed no excess mortality and comparable outcomes between the HD-CTX group and the ATG group. Furthermore, the total medical cost of the HD-CTX group was much less than that of the ATG regimen (18).

Based on these findings, current therapeutic approaches for refractory/relapse SAA include salvage HSCT, a second course of ATG-based IST or HD-CTX. In addition, alemtuzumab (19) and the thrombopoietin receptor agonist (TPO-RA) eltrombopag (20, 21) are also effective in this setting of initial IST failure. However, data to determine the optimal second-line treatment are limited. Therefore, we conducted a retrospective study comparing the long-term efficacy of allo-HSCT with repeated IST as salvage therapy for relapsed/refractory SAA.

## Method

### Patient

The study was performed at the Institute of Hematology and Blood Diseases Hospital Chinese Academy of Medical Sciences. Between January 2007 and December 2022, 69 consecutive patients with relapsed/refractory SAA were enrolled. The detailed inclusion criteria were as follows: (1) age younger than 70 years; (2) diagnosis of SAA or very SAA (VSAA) (22) (congenital bone marrow disorders were excluded); (3) failure of an initial course of ATG plus CsA: TPO-RA was used together with ATG + CsA only in nine patients (eltrombopag in seven patients; hetrombopag in two patients). Refractory SAA was defined as lack of response with persistence of severe pancytopenia at least 3 months after IST. Relapse was considered if the patient had a previous response following IST and once more became transfusion dependent or met criteria for SAA (23); (4) salvaged with allo-HSCT or IST (ATG/CsA or HD-CTX/CsA). Allo-HSCT was indicated if patients were fit enough and had a matched sibling donor (MSD), MUD, or HID. If no suitable donor was available or patients refused HSCT, a second ATG or HD-CTX was applied. The choices of salvage treatment were made by patients and guardians and were also affected by their economic conditions. Patients with paroxysmal nocturnal hemoglobinuria (PNH) clones were also included in this analysis.

### Treatment protocol of HSCT

Patients were conditioned with a FAC or BFAC regimen as previously described (24). The FAC regimen consisted of fludarabine (150 mg/m^2^), CTX (120 mg/kg or 150 mg/kg), and r-ATG (Genzyme, Cambridge, MA, USA, 12.5 mg/kg) or porcine antihuman lymphocyte immunoglobulin (p-ALG, Wuhan Institute of Biological Products, China, 100 mg/kg). The BFAC regimen included intravenous busulfan (Bu, 6.4 mg/kg) on the basis of FAC. Generally, patients with longer disease duration, older age, or PNH clones received the augmented BFAC regimen. Consistent with our previous report (25), graft-versus-host disease (GVHD) prophylaxis regime consisted of CsA/tacrolimus + methotrexate ± mycophenolate mofetil.

### Treatment protocol of IST

In the ATG group, patients were treated with r-ATG 3.0–3.5 mg/kg/day or p-ALG 20 mg/kg/day for five consecutive days (26). In the HD-CTX group, CTX was administered at a dosage of 30 mg/kg/day for four consecutive days (18). Oral CsA was started at an initial dose of 3 mg/kg/day on day 1 and day 11 in the ATG and CTX group, respectively. It was administered for at least 2 years, with subsequent adjustment according to whole blood CsA concentration of 100–200 ng/mL for adults and 100–150 ng/mL for children.

### Definitions

The hematologic response was evaluated at 3, 6, and 12 months after IST. Complete response (CR) was defined as ANC ≥ 1.5×10^9^/L, hemoglobin ≥100 g/L, and platelet count ≥100×10^9^/L. Partial response (PR) was defined as transfusion independence with ANC > 0.5×10^9^/L and no longer met the criteria for severe disease (27). The overall response (OR) included both CR and PR. If blood counts did not meet the criteria of PR or CR, it was assessed as no response (NR).

After HSCT, neutrophil and platelet engraftment, primary and secondary graft failure (GF), and poor graft function (PGF) were defined as previously described (28–30). OS was defined as the time from the initiation of second-line treatment to the last follow-up or death. FFS was defined as survival with response. Death, NR by 6 months and beyond, disease progression requiring clinical intervention, clonal evolution, and relapse were considered treatment failures for IST (31). Death and primary or secondary GF were regarded as failure events for HSCT. Acute GVHD (aGVHD) and chronic GVHD (cGVHD) were graded according to the international criteria (32, 33). GVHD-free, failure-free survival (GFFS) was defined as survival without grades III–IV aGVHD, extensive cGVHD, or treatment failures.

### Statistical analysis

The probabilities of OS, FFS, and GFFS were estimated using the Kaplan–Meier method, with differences compared by the log-rank test. Variables with *p*-values < 0.1 in univariate analysis were evaluated in multivariate analysis using the Cox proportional hazard regression model. In addition, factors including salvage therapy (HSCT or repeated IST), disease course, and status (refractory/relapse) were also incorporated into the model based on previous findings (12, 34). Cumulative incidences (CIs) of engraftment and GVHD were estimated in the competing risk model; death was considered as a competing risk. Differences in the distribution of various parameters were compared using chi-square or Student’s *t*-test as appropriate. *p*-values < 0.05 were considered statistically significant. All statistical analyses were performed using the SPSS version 22.0 and the R software (version 3.4.3).

## Results

### Baseline characteristics

A total of 69 patients were enrolled in the study. Patient characteristics are presented in **Table 1**. In the HSCT group, patients received a transplant from MSD (*n* = 6), MUD (*n* = 7), or HID (*n* = 23). In the second IST group, patients were retreated with ATG (r-ATG, *n* = 7; p-ALG, *n* = 9) or HD-CTX (*n* = 17). Patients in the HSCT cohort were younger (median age at 21 years) than those in the IST cohort (median age at 28 years) (*p* = 0.036). The majority (91.7%, 33/36) of HSCT recipients were refractory SAA, compared to 39.4% (13/33) in the IST group (*p* < 0.001). There were no significant differences in sex ratio, disease duration, severity of disease (SAA/VSAA), or the presence of PNH clones at salvage therapy between the two cohorts.

**Table 1:** Patient characteristics between the HSCT group and the IST group.

	HSCT group (*n* = 36)	IST group (*n* = 33)	*p*-value
Sex, *n* (%)			0.751
Male	15 (41.7)	15 (45.5)	
Female	21 (58.3)	18 (54.5)	
Age at salvage therapy (years), median (range)	21 (3-57)	28 (10-66)	0.036
<20 years, *n* (%)	16 (44.4)	12 (36.4)	0.495
20–40 years, *n* (%)	14 (38.9)	11 (33.3)	0.632
≥40 years, *n* (%)	6 (16.7)	10 (30.3)	0.180
Disease severity, *n* (%)			0.307
SAA	22 (61.1)	24 (72.7)	
VSAA	14 (38.9)	9 (27.3)	
Disease status at salvage therapy, *n* (%)			<0.001
Refractory	33 (91.7)	13 (39.4)	
Relapsed	3 (8.3)	20 (60.6)	
PNH clones at salvage therapy, *n* (%)	8 (22.2)	6 (18.2)	0.677
First IST, *n* (%)			0.104
r-ATG	19 (52.8)	11 (33.3)	
p-ALG	17 (47.2)	22 (66.7)	
Salvage therapy year			0.076
2007–2016	10 (27.8)	16 (48.5)	
2017–2022	26 (72.2)	17 (51.5)	
Median months from diagnosis to second-line therapy (range)			
Refractory SAA	10.0 (4.0–84.5)	10.8 (5.1–31.6)	0.500
Relapsed SAA	84.1 (65.5–99.0)	45.3 (11.3–193.8)	0.264
Median months between first IST and second-line therapy (range)			
Refractory SAA	8.9 (3.0–84.0)	8.9 (4.1–18.3)	0.392
Relapsed SAA	84.0 (65.3–97.9)	39.5 (10.8–164.4)	0.166

HSCT, hematopoietic stem cell transplantation; IST, immunosuppressive therapy; SAA, severe aplastic anemia; VSAA, very severe aplastic anemia; PNH, paroxysmal nocturnal hemoglobinuria; r-ATG, rabbit antithymocyte globulin; p-ALG, porcine antihuman lymphocyte immunoglobulin.

### Outcomes of HSCT

Patient and donor characteristics are shown in **Table 2**. All 36 patients survived for more than 28 days and achieved neutrophil engraftment with a median of 14 (10–24) days. A total of 32 patients (88.9%) achieved platelet engraftment with a median time of 16 (9–152) days. Most patients (31/36) received grafts from peripheral blood (PB), and only 5 received a combination of bone marrow (BM) and PB. The median infused mononuclear cell, CD34+, and CD3+ dose was 10.72 × 10^8^/kg, 3.35 × 10^6^/kg, and 178.11 × 10^6^/kg, respectively. No cases of primary or secondary GF were observed. One patient developed primary PGF, but refused further intervention and died 8.6 months post-transplant. One patient experienced secondary PGF, received CD34-selected PB stem cells from the original donor, and achieved complete trilineage recovery at 7 months after boost infusion. At 3, 6, and 12 months after HSCT, 63.9%, 83.3%, and 86.1% of the recipients had achieved normal blood routine, respectively.

**Table 2:** Characteristics of 36 patients who underwent salvage HSCT.

Variable	*N* = 36
ECOG score pre-HSCT, *n* (%)	
0–1	27 (75.0)
≥2	9 (25.0)
Conditioning regimen, *n* (%)	
Bu + Flu + CTX + ATG	24 (66.7)
Flu + CTX + ATG	12 (33.3)
Donor age (years), median (range)	31 (11–54)
Donor type, *n* (%)	
MSD	6 (16.7)
MUD	7 (19.4)
HID—Parent	11 (30.6)
HID—Child	5 (13.9)
HID—Sibling	7 (19.4)
Donor–recipient sex match, *n* (%)	
Male–male	5 (13.9)
Male–female	15 (41.7)
Female–male	11 (30.5)
Female–female	5 (13.9)
ABO match of donor to recipient, *n* (%)	
Match	21 (58.3)
Major mismatch	7 (19.4)
Minor mismatch	6 (16.7)
Major and minor mismatch	2 (5.6)
Stem cell source, *n* (%)	
PB	31 (86.1)
BM+PB	5 (13.9)
MNC, 10^8^/kg, median (range)	10.72 (5.70–23.10)
CD34+ cells, 10^6^/kg, median (range)	3.35 (2.07–10.40)
CD3+ cells, 10^6^/kg, median (range)	178.11 (73.90–412.98)
28-day neutrophil engraftment, *n* (%)	36 (100)
28-day platelet engraftment, *n* (%)	32 (88.9)
Neutrophil engraftment, d, median (range)	14 (10–24)
Platelet engraftment, d, median (range)	16 (9–152)
Median time no transfusion dependence, days (range)	
RBC	18 (0–155)
PLT	19 (6–167)
Infection post-transplantation, *n* (%)	
CMV viremia	19 (52.8)
EBV viremia	6 (16.7)
Pulmonary infection	5 (13.9)
Bacteremia	2 (5.6)
aGVHD	
100-day II–IV aGVHD	36.1% ± 0.7%
100-day III–IV aGVHD	13.9% ± 0.3%
cGVHD	
4-year mild to severe cGVHD	36.2% ± 0.7%
4-year moderate to severe cGVHD	14.9% ± 0.4%

Bu, busulfan; Flu, fludarabine; CTX, cyclophosphamide; ATG, antithymocyte globulin; MSD, matched sibling donor; MUD, matched unrelated donor; HID, haploidentical donor; PB, peripheral blood; BM, bone marrow; MNC, mononuclear cell; CMV, cytomegalovirus; EBV, Epstein–Barr virus; aGVHD, acute graft-versus-host disease; cGVHD, chronic graft-versus-host disease.

The CI of grades II–IV and III–IV aGVHD on day +100 was 36.1% ± 0.7% and 13.9% ± 0.3%, respectively. A total of 33 patients survived for more than 100 days and were evaluable for cGVHD. The 4-year CI of cGVHD was 36.2% ± 0.7%, and that of moderate to severe cGVHD was 14.9% ± 0.4%. In univariate analysis, patients with higher graft CD34+ cell infusion (>3.35×10^6^/kg) had higher rates of extensive cGVHD (32.8% vs. 5.6%, *p* = 0.045). No other factor was identified on the incidence of GVHD.

A total of 29 patients are alive with a median follow-up of 45.6 (5.9–166.9) months. The causes of death included severe pneumonia (*n* = 3), intracranial hemorrhage (*n* = 1), *Aeromonas hydrophila* bacteremia (*n* = 1), poor graft function (*n* = 1), and suicide (*n* = 1).

### Outcomes of repeated IST

The characteristics of patients and outcomes of repeated IST are summarized in **Table 3**. The median interval between the first and second course of IST was 8.9 (4.1–18.3) months for refractory setting. The median time from the initial ATG to relapse was 36.0 (5.0–132.0) months; the median time between the first and second course of IST was 39.5 (10.8–164.4) months for relapse setting. At 3 months after the initiation of the second IST, response was observed in 12 patients, including 11 with PR and 1 with CR. By 6 months, 29 cases were evaluated, with 4 achieving CR and 15 achieving PR. The ORR among all patients was 57.6% (19/33), being higher in relapsed patients than in those with refractory SAA (65.0% vs. 46.2%), although the difference was not statistically significant due to the limited patient cohort (*p* = 0.284). In addition, patients salvaged with repeated IST pre- or post-2017 showed comparable response rates (68.8% vs. 47.1%, *p* = 0.208). Ten patients improved between 6 and 12 months, and the ORR was 60.6% (20/33) at 12 months, including 13 CR and 7 PR. Hematologic responses in the second ATG group and HD-CTX group showed no significant differences in ORRs at 3 months (37.5% vs. 35.3%, *p* = 0.895), 6 months (50.0% vs. 64.7%, *p* = 0.393), and 12 months (56.3% vs. 64.7%, *p* = 0.619) (**Supplementary Table 1**).

**Table 3:** Patient characteristics and hematologic responses in the repeated IST group.

	Refractory SAA (*n* = 13)	Relapsed SAA (*n* = 20)	*p*-value
Sex, *n* (%)			0.948
Male	6 (46.2)	9 (45.0)	
Female	7 (53.8)	11 (55.0)	
Age at diagnosis (years), median (range)	19 (9–60)	28 (10–62)	0.121
Age at second IST (years), median (range)	19 (10–61)	33 (11–66)	0.037
Disease severity, *n* (%)			0.006
SAA	6 (46.2)	18 (90.0)	
VSAA	7 (53.8)	2 (10.0)	
First IST, *n* (%)			0.208
r-ATG	6 (46.2)	5 (25.0)	
p-ALG	7 (53.8)	15 (75.0)	
Second IST, *n* (%)			0.014
r-ATG	3 (23.1)	4 (20.0)	
p-ALG	0	9 (45.0)	
HD-CTX	10 (76.9)	7 (35.0)	
Interval from diagnosis to first IST, months, median (range)	1.0 (0.3–18.2)	1.0 (0.3–29.4)	0.788
Interval between first and second IST, months, median (range)	8.9 (4.1–18.3)	39.5 (10.8–164.4)	0.001
Interval from diagnosis to second IST, months, median (range)	10.8 (5.1–31.6)	45.3 (11.3–193.8)	0.004
3-month OR, *n* (%)	5 (38.5)	7 (35.0)	0.840
6-month OR, *n* (%)	6 (46.2)	13 (65.0)	0.284
12-month OR, *n* (%)	6 (46.2)	14 (70.0)	0.171
Best response ever reached, *n* (%)			
CR	5 (38.5)	10 (50.0)	0.515
OR	6 (46.2)	14 (70.0)	0.171
Follow-up among alive patients, months, median (range)	89.4 (18.7–199.2)	75.1 (19.9–133.3)	0.299

IST, immunosuppressive therapy; SAA, severe aplastic anemia; VSAA, very severe aplastic anemia; r-ATG, rabbit antithymocyte globulin; p-ALG, porcine antihuman lymphocyte immunoglobulin; HD-CTX, high-dose cyclophosphamide; OR, overall response; CR, complete response.

The median follow-up was 81.6 (18.7–199.2) months among alive patients from the start of second IST. A total of six patients died, and causes of death included pulmonary infection (*n* = 3) and central nervous system infection (*n* = 1). Moreover, two patients received a second course of ATG from the same course and died of severe allergic reaction within 1 week. Two CR patients relapsed 43 and 59 months again, and successfully rescued with the third IST. No secondary clonal disorders were observed.

### Survival outcomes

**Figure 1:**
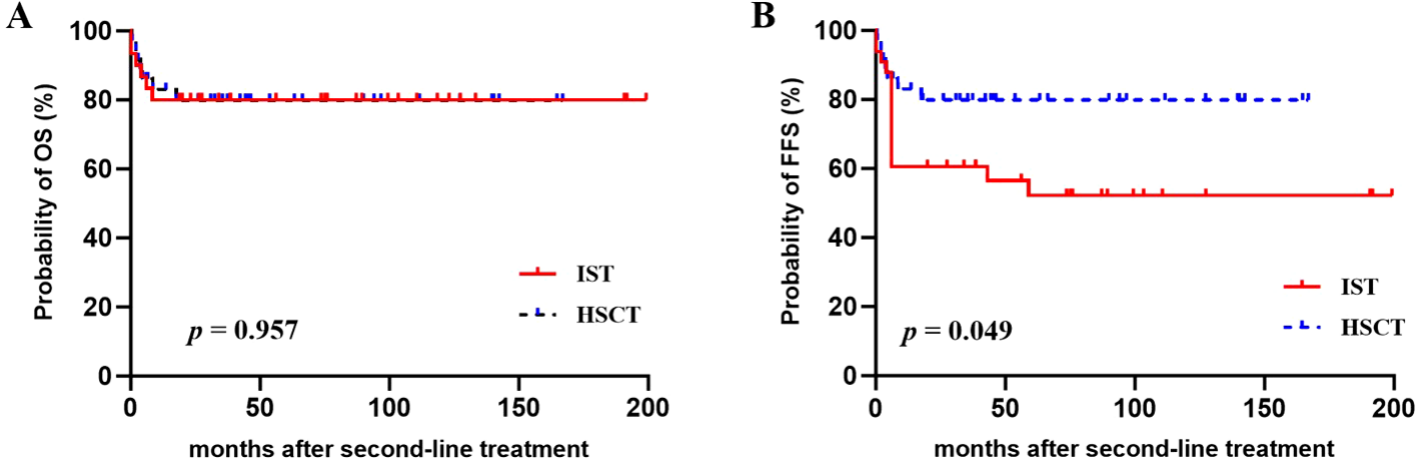
**(A)** Overall survival (OS) and **(B)** failure-free survival (FFS) after second-line treatments with repeated IST or salvage HSCT.

The estimated 4-year OS and FFS for the entire cohort were 80.0% ± 5.0% and 68.3% ± 5.8%, respectively. The 4-year CI of treatment-related mortality (TRM) was 20.0% ± 5.0%. In univariate analysis, younger patients (≤35 years) demonstrated better FFS (77.2% ± 6.1% vs. 43.7% ± 11.9%, *p* = 0.007) and a trend toward improved OS (85.5% ± 5.1% vs. 63.7% ± 11.9%, *p* = 0.057). Compared with repeated IST, salvage allo-HSCT offered a comparable OS (79.8% ± 6.8% vs. 80.0% ± 7.3%, *p* = 0.957) but a significantly higher FFS (79.8% ± 6.8% vs. 56.6% ± 8.8%, *p* = 0.049) (**Figure 1**). Further analysis revealed that the FFS of HSCT was clearly better than that of second ATG (79.8% vs. 49.2%, *p* = 0.018), but comparable to that of HD-CTX (79.8% vs. 64.7%, *p* = 0.295). Multivariate analysis identified age ≤ 35 years as a favorable factor for both the OS [hazard ratio (HR) 0.313, 95% CI 0.102–0.961] and FFS (HR 0.358, 95% CI 0.144–0.890). In addition, the choice of allo-HSCT was an independent factor for superior FFS (HR 0.355, 95% CI 0.128–0.985) (**Table 4**).

**Table 4:** Multivariate analysis of risk factors associated with survival outcomes.

Risk factor	Overall survival		Failure-free survival	
	Hazard ratio (95% CI)	*p*-value	Hazard ratio (95% CI)	*p*-value
Age ≤ 35 years vs. > 35 years	0.313 (0.102–0.961)	0.042	0.358 (0.144–0.890)	0.027
HSCT vs. repeated IST	0.778 (0.215–2.810)	0.701	0.355 (0.128–0.985)	0.047
Refractory SAA vs. relapsed SAA	2.106 (0.377–11.779)	0.396	0.344 (0.107–1.106)	0.073
SAA course (≤24 vs. >24 months)	1.266 (0.303–5.291)	0.747	1.676 (0.588–4.775)	0.334

HSCT, hematopoietic stem cell transplantation; IST, immunosuppressive therapy; SAA, severe aplastic anemia.

We further analyzed survival outcomes for the HSCT group and the IST group, respectively. In the HSCT cohort (**Supplementary Table 2**), older patients (>35 years) showed a significantly lower 4-year OS (38.1% vs. 89.2%, *p* = 0.002) and FFS (38.1% vs. 89.2%, *p* = 0.002) when compared with younger patients. In addition, OS and FFS were worse in recipients conditioned with the BFAC regimen than with the FAC regimen. It was noteworthy that compared to the FAC regimen, patients in the BFAC cohort were older (26 years vs. 14 years, *p* = 0.022) and had a longer disease course (13 months vs. 9 months, *p* = 0.039), which might contribute to their inferior survival outcomes. The GFFS was also calculated in the HSCT group, which was considered to be an alternative marker of quality of survival. The estimated 4-year GFFS was 71.4% ± 7.7%; poor GFFS was observed in MUD (42.9%) transplants as compared to MSD (83.3%) and HID (77.4%) transplants (*p* = 0.049), and also in older patients (>35 years) (38.1% vs. 78.8%, *p* = 0.052). Because of the limited HSCT cohort size, we did not perform further multivariate analysis. In the IST cohort, none of the assessed variables [age, disease course, disease status (refractory/relapse), retreated with second ATG or HD-CTX, and salvage therapy year (pre- or post-2017)] were detected to affect the survival.

### Analysis of subgroup

**Figure 2:**
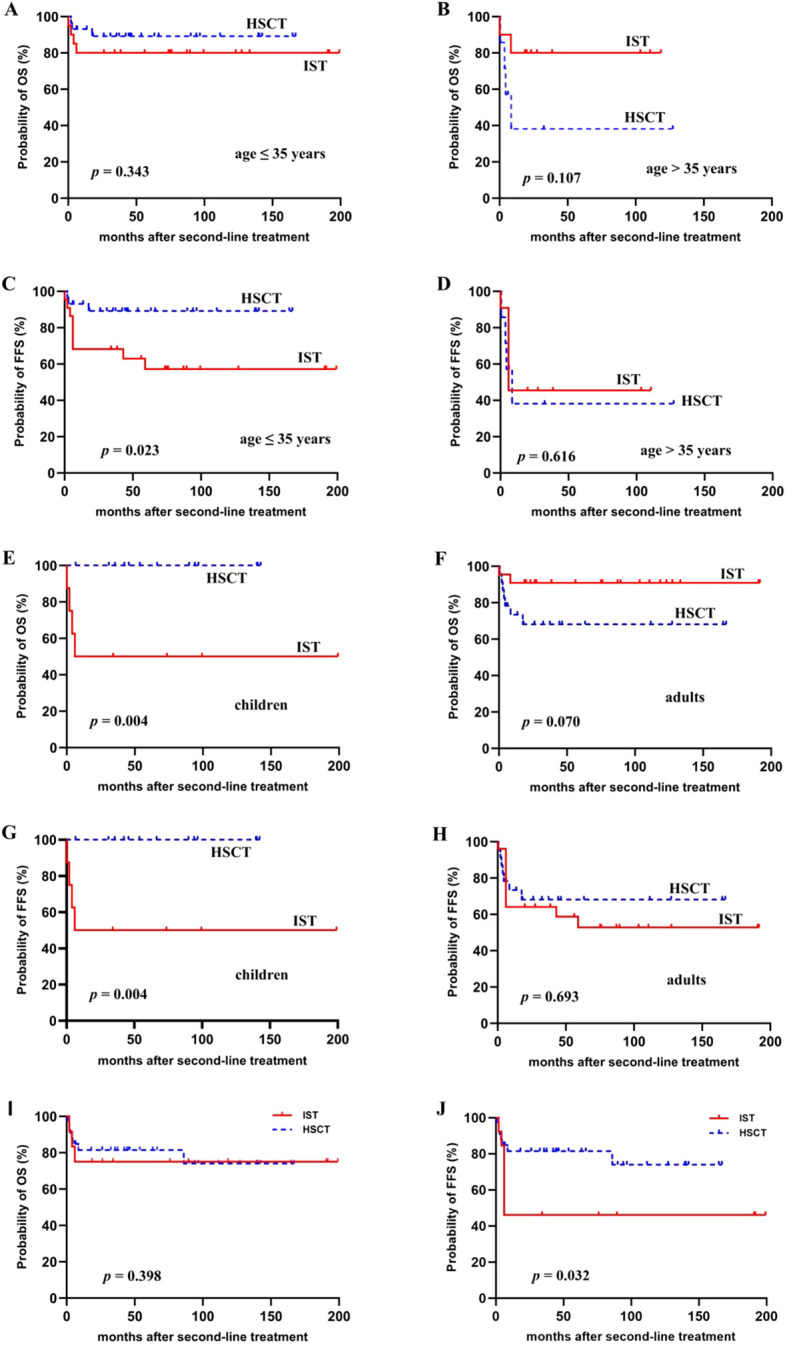
Subgroup analysis of survival outcomes following repeated IST or salvage HSCT. **(A–D)** The overall survival (OS) and failure-free survival (FFS) for patients with two age stratifications (≤35 years, >35 years). **(E–H)** The OS and FFS for children and adults, respectively. **(I, J)** The OS and FFS in patients with refractory SAA.

As we identified age ≤ 35 years as an independent prognostic factor, we stratified patients into two age groups: ≤35 years and >35 years (**Figures 2A–D**). For patients aged ≤35 years, salvage HSCT provided a similar 4-year OS (89.2% ± 5.9% vs. 80.8% ± 8.9%, *p* = 0.343) but a far better FFS (89.2% ± 5.9% vs. 62.9% ± 10.5%, *p* = 0.023) than a second IST. For patients aged >35 years, no significant differences in survival were observed between the two salvage therapies.

In addition, considering our study included both children (*n* = 21) and adults (*n* = 48), we analyzed survival outcomes between the two groups (**Figures 2E–H**). The 4-year OS was 81.0% ± 8.6% for children and 79.6% ± 6.1% for the adult cohort (*p* = 0.960). The 4-year FFS was 81.0% ± 8.6% for children and 62.5% ± 7.4% for the adult cohort (*p* = 0.167). Of note, children in the HSCT group were all alive without treatment failures, yielding a significantly higher 4-year OS (100% vs. 50.0% ± 17.7%, *p* = 0.004) and FFS (100% vs. 50.0% ± 17.7%, *p* = 0.004) than children in the IST group. For adults, however, outcomes of transplants and repeated IST were not significantly different.

We also conducted subgroup analysis in 46 patients with refractory SAA, and found this group benefited more from HSCT than repeated IST. At 6 months after second-line treatment, the CR rate was 81.8% in the HSCT cohort, while it was only 7.7% in the IST cohort (*p* < 0.001). The 4-year OS was not statistically different (81.5% vs. 69.2%, *p* = 0.398); however, the FFS was far better for patients salvaged with HSCT than with IST (81.5% vs. 46.2%, *p* = 0.032) (**Figures 2I, J**).

## Discussion

Patients with refractory/relapse SAA are at increased risk of death from infection and hemorrhage and later clonal evolution. Outcomes remain poor for them and the best option is not consensual. In the current study, we conducted a long-term follow-up of 69 patients with SAA who were rescued with HSCT or repeated intensified IST after failure of the first ATG-base IST. Compared with repeated IST, we observed that HSCT provided a superior FFS in both univariate and multivariate analysis. Subgroup analyses revealed that younger patients (age ≤ 35 years) and patients with refractory SAA benefited more from HSCT.

In our repeated IST cohort, two patients received a second ATG from the same source and died of severe allergic reactions within 1 week. In a prospective multicenter trial (35), likewise, 3 out of 21 patients developed an anaphylactoid reaction to the same source h-ATG and could not complete the second IST. Furthermore, Tichelli et al. found that serum sickness occurred earlier after repeated ATG as compared to initial exposure (36). Therefore, anaphylactic reactions and serum sickness are worth noting in the second course of ATG. In the HD-CTX group, however, patients showed better tolerability with no excess mortality, which might be attributed to the reduction dose of CTX and the deferral administration of CsA in our modified regimen. Although the FFS of second ATG was inferior to salvage HSCT, the other regimen of IST, HD-CTX plus CsA, achieved an FFS that was comparable to HSCT. However, owing to the limited numbers, the results need to be confirmed in a larger study.

For patients who have relapsed after the initial IST, a prior response implies an immune-mediated pathogenetic mechanism, and thus, 50%–65% of them can be successfully salvaged with a second IST (10). For patients with refractory SAA, the lack of response may due to non-immune-based pathophysiology, extreme hematopoietic stem cell exhaustion, or inadequate immunosuppression (10, 37). Several studies have reported unsatisfactory responses to a further course of IST in a refractory setting (12, 19, 35, 38). In our IST cohort, the response rate at 6 months was 57.6%, and it was also lower in refractory patients than in relapsed patients (46.2% vs. 65.0%). In the subgroup analysis of refractory SAA, we found that repeated IST provided a significantly lower CR rate at 6 months (7.7% vs. 81.8%, *p* < 0.001) and FFS at 4 years (46.2% vs. 81.5%, *p* = 0.032) when compared with HSCT. Therefore, it is reasonable to consider HSCT instead of a second IST for refractory patients.

Favorable outcomes of HSCT post-IST failure have been investigated in many studies. Kosaka and colleagues compared the efficacy of repeated IST and alternative donor HSCT in children who failed previous IST and found that HSCT provided a better chance of FFS at 5 years than a second course of IST (83.9% vs. 9.5%, *p* = 0.001) (35). The long-term outcomes in the HSCT cohort of our study were also encouraging, with an estimated 4-year OS and FFS of 79.8% ± 6.8%. Notably, all children in the HSCT cohort were alive without treatment failures, showing a 4-year OS and FFS of 100%, which is significantly higher than that of children in the IST cohort. In addition, HSCT offered a superior FFS than repeated IST for patients aged ≤35 years, while no such survival advantage was observed in patients aged >35 years. These results suggested that younger patients, especially children, benefited more from HSCT than a second IST.

Graft failure is a major concern for transplant patients with refractory/relapse SAA, as they are often heavily transfused and have a longer course of disease. In our research, over half (63.9%) of the recipients underwent alternative donor HSCT. Of note, no primary or secondary graft failure was observed. The encouraging outcome might be partly attributed to the application of the intensified BFAC conditioning regimen in the majority (24/36) of patients. As previously reported (39, 40), adding BU or low-dose total body irradiation for augmented conditioning facilitated engraftment in SAA patients. Hashem et al. also showed that application of a more intense conditioning was successful in overcoming the high rates of GF (8). However, as we mentioned above, recipients conditioned with the BFAC regimen were older and had a longer disease course than recipients conditioned with the FAC regimen. Several studies (41–43) have demonstrated that older age and a longer SAA history were negative predictors of survival. Consequently, patients in the BFAC group showed inferior survival, which was consistent with our previous report (24).

Several studies (44, 45) have demonstrated higher rates of GVHD in the salvage cohort compared to frontline MSD-HSCT. A significantly high proportion of alternative donor HSCT in the salvage setting might contribute to this disparity. Our results showed acceptable GVHD; the 100-day grades II–IV aGVHD was 36.1%, and 4-year cGVHD was 36.2%. The rates were comparable to our previous studies involving HID-HSCT for SAA (24, 46), reported II–IV aGVHD rates of 35%–38.4%, and cGVHD rates of 23%–35.3%. The 4-year GFFS was 71.4% ± 7.7%, which was not inferior to our recent data (47) reporting the rates of 61.2%–67.6% in 260 AA patients. In conclusion, our data presented the long-term outcomes of allo-HSCT and repeated IST in the salvage setting for patients with refractory/relapse SAA. Compared with IST, HSCT exhibited clear advantages in rapid complete hematopoietic recovery and superior FFS, especially in younger patients (≤35 years) and refractory setting. Therefore, for these patients, salvage HSCT may be more preferable than a second course of IST. We acknowledge several limitations of our study, including its retrospective nature and a relatively small sample size from a single center. Further prospective, multicenter studies in large cohorts are warranted to validate our results.

## Data Availability

The original contributions presented in the study are included in the article/supplementary material. Further inquiries can be directed to the corresponding authors.
