# Supplementary material for: Comparison of hematopoietic stem cell transplantation and repeated intensified immunosuppressive therapy as second-line treatment for relapsed/refractory severe aplastic anemia
Source: Front Immunol. 2024 Aug 16;15:1425076. doi: 10.3389/fimmu.2024.1425076 (PMC11361938; doi:10.3389/fimmu.2024.1425076)
Supplement: Supplementary file 1 [file Table1.docx]

**Supplementary Table 1** Hematologic responses and survival outcomes between the second ATG and HD-CTX group

|  | **Second ATG group**  **(n = 16)** | **HD-CTX group**  **(n = 17)** | ***P* Value** |
| --- | --- | --- | --- |
| 3 months, n (%) |  |  |  |
| CR | 1 (6.3) | 0 |  |
| PR | 5 (31.3) | 6 (35.3) |  |
| NR | 8 (50.0) | 10 (58.8) |  |
| OR | 6 (37.5) | 6 (35.3) | 0.895 |
| Dead | 2 (12.5) | 1 (5.9) |  |
| 6 months, n (%) |  |  |  |
| CR | 2 (12.5) | 2 (11.8) |  |
| PR | 6 (37.5) | 9 (52.9) |  |
| NR | 5 (31.3) | 5 (29.4) |  |
| OR | 8 (50.0) | 11 (64.7) | 0.393 |
| Dead | 3 (18.8) | 1 (5.9) |  |
| 12 months, n (%) |  |  |  |
| CR | 6 (37.5) | 7 (41.2) |  |
| PR | 3 (18.8) | 4 (23.5) |  |
| NR | 3 (18.8) | 4 (23.5) |  |
| OR | 9 (56.3) | 11 (64.7) | 0.619 |
| Dead | 4 (25.0) | 2 (11.8) |  |
| Best response ever reached, n (%) |  |  |  |
| CR | 7 (43.8) | 8 (47.1) | 0.849 |
| OR | 9 (56.3) | 11 (64.7) | 0.619 |
| 4-year OS | 71.4 ± 12.1% | 87.5 ± 8.3% | 0.271 |
| 4-year FFS | 49.2 ± 12.7% | 64.7 ± 11.6% | 0.234 |

ATG, antithymocyte globulin; HD-CTX, high-dose cyclophosphamide; CR, complete response; PR, partial response; NR, no response; OR, overall response; OS, overall survival; FFS, failure-free survival.
