# Supplementary material for: Comparison of hematopoietic stem cell transplantation and repeated intensified immunosuppressive therapy as second-line treatment for relapsed/refractory severe aplastic anemia
Source: Front Immunol. 2024 Aug 16;15:1425076. doi: 10.3389/fimmu.2024.1425076 (PMC11361938; doi:10.3389/fimmu.2024.1425076)
Supplement: Supplementary file 2 [file Table2.docx]

**Supplementary Table 2** Factors associated with outcomes in the HSCT cohort in univariate analysis

|  | **No.** | **4-year OS**  **(%)** | ***P* Value** | **4-year FFS**  **(%)** | ***P* Value** | **4-year GFFS (%)** | ***P* Value** |
| --- | --- | --- | --- | --- | --- | --- | --- |
| Sex |  |  | 0.895 |  | 0.895 |  | 0.112 |
| Male | 15 | 78.8 ± 11.0% |  | 78.8 ± 11.0% |  | 58.3 ± 13.2% |  |
| Female | 21 | 80.7 ± 8.7% |  | 80.7 ± 8.7% |  | 80.7 ± 8.7% |  |
| Age (years) |  |  | 0.002 |  | 0.002 |  | 0.052 |
| ≤ 35 | 29 | 89.2 ± 5.9% |  | 89.2 ± 5.9% |  | 78.8 ± 7.7% |  |
| > 35 | 7 | 38.1 ± 19.9% |  | 38.1 ± 19.9% |  | 38.1 ± 19.9% |  |
| Disease severity |  |  | 0.743 |  | 0.743 |  | 0.700 |
| SAA | 22 | 75.9 ± 9.4% |  | 75.9 ± 9.4% |  | 66.3 ± 10.4% |  |
| VSAA | 14 | 84.4 ± 10.2% |  | 84.4 ± 10.2% |  | 77.1 ± 11.7% |  |
| SAA course (months)  < 10.5 |  |  | 0.723 |  | 0.723 |  | 0.920 |
| < 10.5 | 18 | 76.2 ± 10.5% |  | 76.2 ± 10.5% |  | 70.5 ± 11.2% |  |
| ≥ 10.5 | 18 | 83.3 ± 3.8% |  | 83.3 ± 3.8% |  | 72.2 ± 10.6% |  |
| ECOG pre-HSCT |  |  | 0.060 |  | 0.060 |  | 0.370 |
| 0-1 | 28 | 85.3 ± 6.8% |  | 85.3 ± 6.8% |  | 74.8 ± 8.3% |  |
| ≥ 2 | 8 | 62.5 ± 17.1% |  | 62.5 ± 17.1% |  | 62.5 ± 17.1% |  |
| Active infection pre-HSCT |  |  | 0.892 |  | 0.892 |  | 0.561 |
| Yes | 10 | 80.0 ± 12.6% |  | 80.0 ± 12.6% |  | 80.0 ± 12.6% |  |
| No | 26 | 79.8 ± 8.1% |  | 79.8 ± 8.1% |  | 68.1 ± 9.4% |  |
| Ferritin pre-HSCT (ug/L) |  |  | 0.295 |  | 0.295 |  | 0.478 |
| < 2000 | 22 | 85.2± 8.0% |  | 85.2± 8.0% |  | 76.0 ± 9.5% |  |
| ≥ 2000 | 14 | 71.4± 12.1% |  | 71.4± 12.1% |  | 64.3± 12.8% |  |
| Conditioning regimen |  |  | 0.038 |  | 0.038 |  | 0.071 |
| Bu+Flu+CTX+ATG | 24 | 68.9 ± 9.9% |  | 68.9 ± 9.9% |  | 60.3 ± 10.5% |  |
| Flu+CTX+ATG | 12 | 100% |  | 100% |  | 91.7 ± 8.0% |  |
| Donor type |  |  | 0.732 |  | 0.732 |  | 0.049 |
| MSD | 6 | 83.3 ± 15.2% |  | 83.3 ± 15.2% |  | 83.3 ± 15.2% |  |
| MUD | 7 | 71.4 ± 17.1% |  | 71.4 ± 17.1% |  | 42.9 ± 18.7% |  |
| HID | 23 | 81.8 ± 8.3% |  | 81.8 ± 8.3% |  | 77.4 ± 8.9% |  |
| Donor-recipient sex match |  |  | 0.312 |  | 0.312 |  | 0.994 |
| Female-male | 11 | 90.9 ± 8.7% |  | 90.9 ± 8.7% |  | 72.7 ± 13.4% |  |
| others | 25 | 75.2 ± 8.8% |  | 75.2 ± 8.8% |  | 71.1 ± 9.3% |  |
| CD34+ cells, 10^6^/kg |  |  | 0.591 |  | 0.591 |  | 0.467 |
| ≤ 3.35 | 18 | 83.3 ± 8.8% |  | 83.3 ± 8.8% |  | 77.8 ± 9.8% |  |
| > 3.35 | 18 | 75.8 ± 10.8% |  | 75.8 ± 10.8% |  | 72.2 ± 10.6% |  |
| HSCT year |  |  | 0.322 |  | 0.322 |  | 0.878 |
| 2007-2016 | 10 | 90.0 ± 9.5% |  | 90.0 ± 9.5% |  | 70.0 ± 14.5% |  |
| 2017-2022 | 26 | 76.3 ± 8.5% |  | 76.3 ± 8.5% |  | 72.4 ± 8.9% |  |

OS, overall survival; FFS, failure-free survival; GFFS, graft-versus-host disease-free, failure-free survival; SAA, severe aplastic anemia; VSAA, very severe aplastic anemia; Bu, busulfan; Flu, fludarabine; CTX, cyclophosphamide; ATG, antithymocyte globulin; MSD, matched sibling donor; MUD, matched unrelated donor; HID, haploidentical donor.
